# Revealing different aggregational states of a conjugated polymer in solution by a nanopore sensor†
†Electronic supplementary information (ESI) available. See DOI: 10.1039/c6sc00296j


**DOI:** 10.1039/c6sc00296j

**Published:** 2016-05-05

**Authors:** Bingyuan Guo, Zhiyi Yao, Lei Liu, Hai-Chen Wu

**Affiliations:** a Key Laboratory for Biomedical Effects of Nanomaterials & Nanosafety , Institute of High Energy Physics , Chinese Academy of Sciences , Beijing 100049 , China . Email: haichenwu@ihep.ac.cn ; Fax: +86-10-88235745 ; Tel: +86-10-88235745; b Beijing National Laboratory for Molecular Sciences , Key Laboratory of Analytical Chemistry for Living Biosystems , Institute of Chemistry , Chinese Academy of Sciences , Beijing 100190 , China

## Abstract

The functionalities of conjugated polymers are determined not only by local molecular structure, but also by the mesoscale conformational and morphological states of the polymer chains. Simulation studies have successfully established the connections between molecular structure and conformational states of certain conjugated polymers. However, experimental tools that can accurately discriminate between different conformational and morphological states of conjugated polymers are still scarce. Here, we use a nanopore sensor to analyze different aggregational states of a polythiophene derivative by threading the polymer through the pore under applied potentials. When the fluorescence of the polythiophene is quenched by pH tuning or the presence of Dy^3+^, the UV-vis and fluorescence spectra of the two solutions appear indistinguishable. However, threading the polymer molecules of these two solutions through an α-hemolysin nanopore affords entirely different translocation profiles owing to their different aggregational states. We further substantiate the results by conducting aggregational interconversion experiments and TEM measurements. This work has clearly indicated that nanopores are promising tools for the analysis of aggregational changes of conjugated polymers and may open new avenues for the investigation of aggregational states of biomacromolecules in the context of early disease diagnosis and prognosis.

## Abstract 2

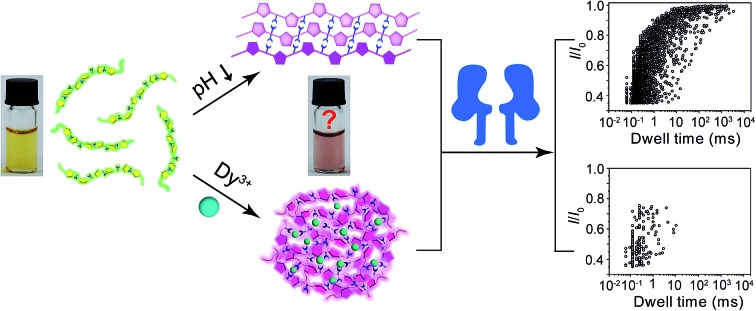
Nanopores are effective and powerful tools for the analysis of conformational and aggregational states of conjugated polymers in solution.

## Introduction

Conjugated polymers have found widespread use in optoelectronic applications including light-emitting diodes,^1^,^2^ field effect transistors,^3^,^4^ solar cells^5^–^8^ and organic lasers;^9^ chemical and biological sensing such as sensitive detection of biomacromolecules (DNA, protein and cell);^10^–^12^ and cell imaging.^13^ Previous studies by experimental and computational scientists have confirmed that the functionalities of conjugated polymers are determined not only by local molecular structure, but also by the mesoscale conformational and morphological states (∼10–100 nm) of the polymer chains.^14^–^16^ The chemical structures of monomers could be readily controlled by design and synthesis; however, the conformational and morphological attributes they induce are much more difficult to characterize. Simulations have succeeded in establishing the connections between molecular structure and various conformational states of certain conjugated polymers.^17^,^18^ But so far, there exist very few experimental tools that can unambiguously discriminate between different conformational and aggregational states of conjugated polymers in solution. Herein, we propose to use a protein nanopore, α-hemolysin (αHL), to probe different conformational states of a polythiophene derivative under varied conditions.

Nanopore sensors have been playing increasingly important roles in analytical science and biophysics in recent years due to their potential implications in DNA sequencing,^19^–^21^ molecular sensing^22^–^24^ and early disease diagnosis.^25^,^26^ Nanopore sensing is a single-molecule technique because when a molecule binds with the nanopore, the ionic current flowing through the nanopore will be modulated and recorded. The frequency of the current events is proportional to the concentration of an analyte, and the combined statistical analyses of the current amplitude change and the dwell time could reveal the analyte's identity. Nanopore sensors were initially developed for the analysis of DNA molecules; but so far there have been few successful examples of applying nanopores for other polymer analyses.^27^–^31^ One notable example is that Kasianowicz and coworkers introduced a two-dimensional method for mass spectrometry in solution based on the interactions between a protein nanopore and poly(ethylene glycol) molecules.^27^ In the present work, we find that different aggregational states of a polythiophene derivative can be discriminated by threading the polymer through αHL under applied potentials. The advantage of the approach could be appreciated by comparing the results with those obtained by UV-vis and fluorescence spectroscopy under the same conditions. We anticipate that this approach may find useful applications in the conformational and aggregational analyses of biomacromolecules in biomedical studies.

## Results and discussion

**Fig. 1:**
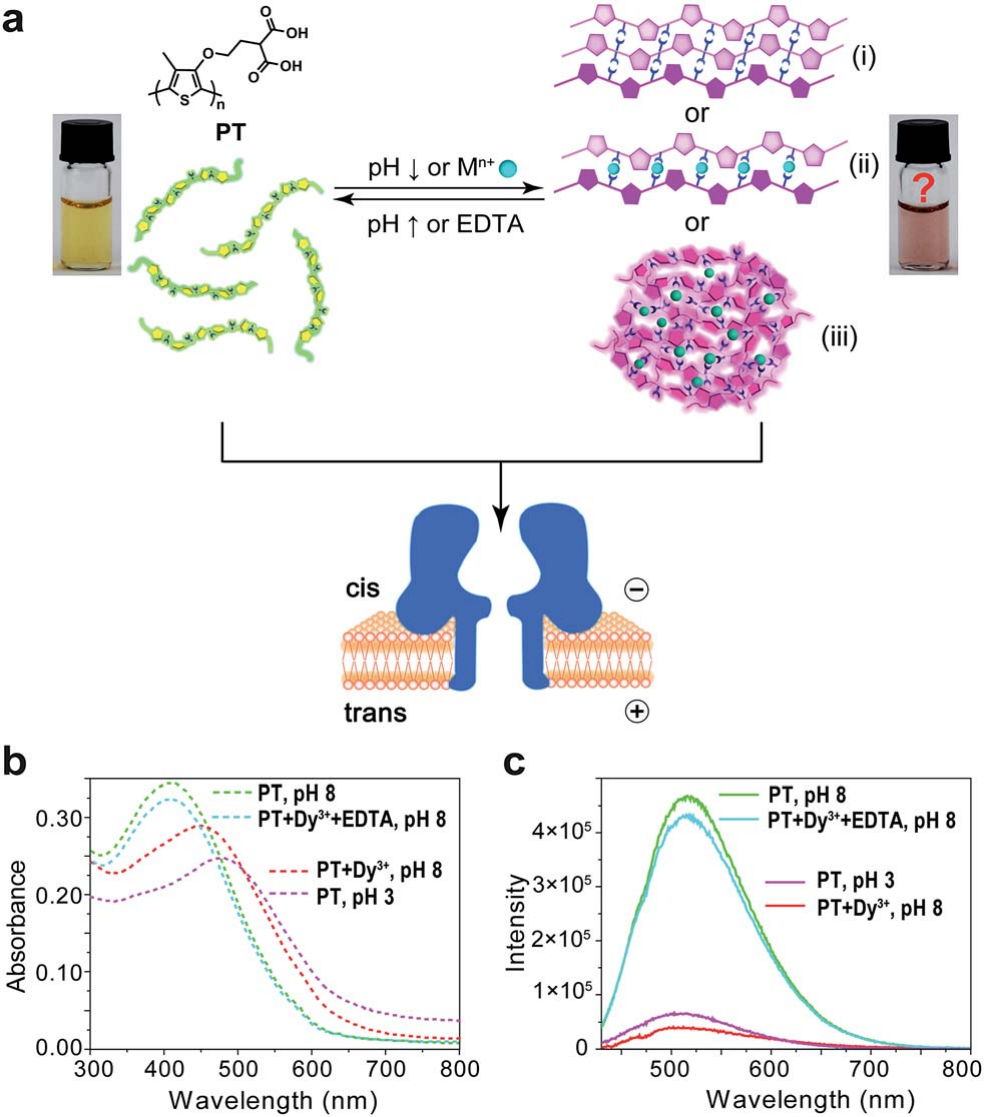
Aggregational switches between dispersed and aggregated states of PT. (a) The aggregational equilibrium of PT in aqueous solution mediated by the adjustment of the solution pH or the presence of metal ions and EDTA. The two pictures represent dispersed and aggregated states of PT, respectively. (b) Absorption spectra (dash lines) of ∼758 nM PT in 10 mM Tris buffer under the following conditions: pH 8 (green); 100 μM Dy^3+^ + 400 μM EDTA, pH 8 (cyan); 100 μM Dy^3+^, pH 8 (red); pH 3 (magenta). (c) Emission spectra (solid lines) of ∼75.8 nM PT in 10 mM Tris buffer under the following conditions: pH 8 (green); 50 μM Dy^3+^ + 100 μM EDTA, pH 8 (cyan); 50 μM Dy^3+^, pH 8 (red); pH 3 (magenta). Excitation wavelength *λ*_ex_ = 410 nm.

Polythiophene is one type of important conjugated polymer that has been used in a wide variety of optoelectronic devices,^32^,^33^ and in particular as chemical sensors.^34^–^36^ Most of the polythiophene-mediated sensing applications are based on the same principle – when water-soluble polythiophene molecules are in dispersed form in solution, they appear yellow and emit strong fluorescence; whereas when the polymers aggregate, either by self assembly or induced by analyte binding, their colour changes to violet or pink along with prominent fluorescence quenching. The polythiophene derivative we used in this work is poly(2-(2-(4-methylthiophen-3-yloxy)-ethyl)malonic acid) (abbreviated as PT), which contains a malonic acid moiety on the side chain of the polymer backbone (Fig. 1a). We had examined the halochromismic behaviour of this molecule^37^ and also employed it for the detection of protamine.^38^ At high pH (8.0–12.0), PT exhibits an absorption maximum at 407 nm, indicative of a random-coiled conformation of the polymer backbone. The solution appears yellow in colour and displays a strong fluorescence emission at 545 nm upon excitation at 410 nm. When the solution pH was adjusted to a lower range (2.0–6.0), the absorption maximum red-shifted to 495 nm and the solution turned pink which was presumed to indicate the formation of an ordered arrangement of PT aggregates. Meanwhile, the fluorescence emission at 545 nm decreased to less than 10% of initial intensity (Fig. 1b and c). This transition process has been corroborated by additional experiments and is similar to what have been observed for other polythiophene sensors.^35^ However, the conformational orders of any PT aggregates have not yet been elucidated experimentally.

During the studies of sensing performance of PT, we found that the presence of various metal ions (Dy^3+^, Ca^2+^, Mg^2+^, Cu^2+^, Co^2+^, Ni^2+^, Zn^2+^, Cd^2+^, Hg^2+^, Mn^2+^) could also cause the transition of PT from random-coiled conformation (yellow) to ordered aggregates (pink). The fluorescence changes were very similar to that of the pH tuning experiments as well (Fig. 1b and c; Dy^3+^ is used as a typical example). From solution colour changes, UV-vis absorption and fluorescence emission spectra, it seems impossible to discriminate between the experimental outcomes caused by pH alteration and by the chelation of metal ions. However, the driving forces for the formation of aggregates under the above two circumstances are distinctly different. In the case of pH tuning, intermolecular hydrogen bonding should be the main factor in assembling the PT molecules; while in the case of the addition of metal ions, metal–anion bonding interactions should be responsible for sticking polymers together. Owing to our experience in nanopore research,^39^–^42^ we decided to investigate the conformational states of the PT aggregates with this single-molecule technique.

**Fig. 2:**
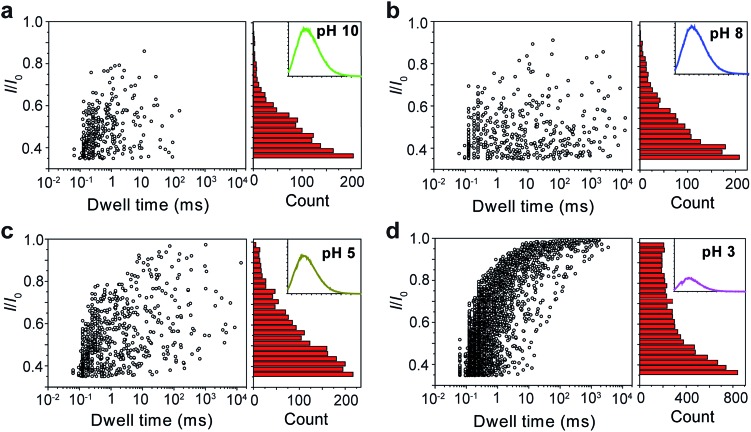
Translocation of PT molecules through αHL under different pH values. (a–d) Left: scatter plots showing current blockades (*I*/*I*_0_) *versus* durations of the translocation events under pH 10.0, 8.0, 5.0, 3.0, respectively. The frequency of PT translocation is ∼1300 ± 280 events per h (a), ∼1600 ± 170 events per h (b), ∼2300 ± 330 events per h (c), ∼13 000 ± 450 events per h (d), respectively. Right: histograms of the current blockades of the translocation events under pH 10.0, 8.0, 5.0, 3.0, respectively. The inset shows the emission spectrum of each PT solution for the translocation studies (for full spectra and conditions, see Fig. S2†). The final concentration of PT is ∼75.8 nM. Scatter plots were constructed using the data points with *I*/*I*_0_ > 0.35 (for data selection criterion, see Fig. S3†) in a 20 min recording trace; current blockade histograms were constructed using the data points in a 60 min trace. Data were acquired in the buffer of 1 M KCl, 10 mM Tris and 0.5 μM EDTA under the pH indicated, with the transmembrane potential held at +100 mV. The number of individual experiments *n* = 3.

The repeating unit of PT has two carboxylic groups which make the polymer negatively charged in the buffer over a wide pH range. This means PT can be threaded through a nanopore under applied potentials similar to DNA translocation. Therefore, we carried out PT translocation experiments through αHL in the buffer of 1 M KCl, 10 mM Tris and 0.5 μM EDTA. All the current events were recorded at a sampling frequency of 100 kHz and filtered with a corner frequency of 10 kHz. After we screened for an optimized transmembrane potential at +100 mV (Fig. S1†), we examined PT translocation through αHL under different pH values (Fig. 2). When the pH of the buffer was decreased from 10.0 to 8.0, there was hardly any change that could be directly observed. Statistical data showed that the frequency of translocation events increased from ∼1300 ± 280 events per h at pH 10.0 to ∼1600 ± 170 events per h at pH 8.0 (Fig. 2a and b). When the pH was further lowered to 5.0, the frequency of events increased to ∼2300 ± 330 events per h with slightly deeper current blockades (larger *I*/*I*_0_) (Fig. 2c). However, drastic changes were observed when the pH was further tuned down to 3.0. First, the frequency of translocation events suddenly increased to ∼13 000 ± 450 events per h. Second, there was a prominent increase of the portion of large current blockage in the range of *I*/*I*_0_ = 0.7–1.0 (Fig. 2d). In theory, tuning pH from 10.0 to 3.0 would largely protonate the malonic acid moieties on the side chain of PT (for malonic acid, p*K*_a_1__ = 2.85 and p*K*_a_2__ = 5.70). This effect alone would decrease the translocation of PT through αHL under the same voltage. But the experimental results supported the opposite conclusion, which could be explained by the changes of the charge selectivity of the protein pore^43^ and the aggregational states of PT molecules induced by the intermolecular hydrogen-bonding. Fluorescence spectra were also acquired under different pH values which were in supportive of PT aggregation upon pH tuning down (Fig. S2†).

**Fig. 3:**
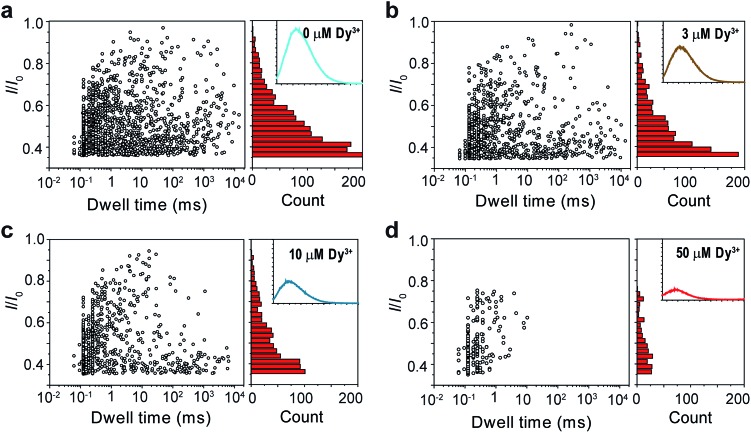
Translocation of PT through αHL in the presence of different concentrations of Dy^3+^. (a–d) Left: scatter plots showing current blockades (*I*/*I*_0_) *versus* durations of the translocation events in the presence of 0, 3, 10, 50 μM Dy^3+^, respectively. The frequency of PT translocation is ∼1600 ± 170 events per h (a), ∼1000 ± 140 events per h (b), ∼800 ± 140 events per h (c), ∼200 ± 20 events per h (d), respectively. Right: histograms of the current blockades of the translocation events in the presence of 0, 3, 10, 50 μM Dy^3+^, respectively. The inset shows the emission spectrum of each PT solution for the translocation studies (for full spectra and conditions, see Fig. S6†). The final concentration of PT is ∼75.8 nM. Both scatter plots and current blockade histograms were constructed using the data points with *I*/*I*_0_ > 0.35 in a 60 min recording trace. Data were acquired in the buffer of 1 M KCl, 10 mM Tris and 0.5 μM EDTA, pH 8.0 in the presence of Dy^3+^ (concentrations indicated), with the transmembrane potential held at +100 mV. The number of individual experiments *n* = 3.

It was mentioned above that metal ions could also induce the aggregation of PT molecules. Thus, we continued to study the PT translocation through αHL in the presence of metal ions. However, when we used the buffer that contained 1 M KCl as the electrolyte for the single-channel recording experiments, we found that addition of Ca^2+^ up to 100 μM barely affected the translocation of PT at pH 10.0 (Fig. S4†). Accordingly, when we measured the fluorescence spectra of PT under those conditions, we found that the quenching effects of most divalent metal ions were lost. This is very likely because the duplex structure formed between PT and divalent metal ions through metal–anion interactions collapses in high ionic electrolyte solution (Fig. 1a(ii)). We also tried to lower the KCl concentration to reduce the influence of the electrolyte, but it was found that PT translocation events nearly completely disappeared under low salt concentrations (Fig. S5†).^44^ We presumed that rare earth metal ions such as Dy^3+^, which has much higher binding affinity toward malonic acid groups than the divalent metal ions,^45^ might afford different results. Interestingly, after different concentrations of Dy^3+^ were added in the PT solution at pH 8.0 for translocation studies, we observed striking differences between the frequencies of translocation events (Fig. 3). As the concentration of Dy^3+^ in solution was increased, the PT translocation was severely hindered. When the final concentration of Dy^3+^ reached 50 μM in solution, the frequency of translocation events dropped to ∼200 ± 20 events per h. Fluorescence spectrum was also obtained under this condition which clearly substantiated the quenching ability of Dy^3+^ (Fig. 3d).

The results in Fig. 2 and 3 clearly demonstrated that the aggregational changes of PT in solution induced by pH tuning and the addition of Dy^3+^ could be unambiguously distinguished by a nanopore sensor. When the pH of the solution is tuned from 10.0 to 3.0, PT molecules aggregate to form oligomers driven by hydrogen-bonding and produce deep current blockage when translocated through αHL (Fig. 1(i)). While in the presence of Dy^3+^, the malonic acid groups on the side chains of PT molecules chelate with Dy^3+^ to induce PT aggregation and further crosslink to form globules in solution (Fig. 1(iii)). The size of the globules is too large to enter the *cis* nanocavity of αHL and thus the frequency of the translocation events drastically decreases. However, the altered polymer–nanopore interactions under the above conditions may also account for the results observed in Fig. 2 and 3. Therefore, we carried out control experiments using a short DNA strand, poly(dT)_30_, to investigate the changes of the DNA translocation profiles under different pH conditions (Fig. S7†) or in the presence of different concentrations of Dy^3+^ (Fig. S8†). The results showed that when the pH of the buffer solution was tuned from 10.0 to 3.0, the frequency of DNA translocation also markedly increased, which probably resulted from the protonation of aspartate and glutamate residues in the αHL nanopore.^46^ There was also a notable change in DNA blockage amplitude under low pH, but the increase is less significant than that of PT (Fig. 2d and S7d†). Whereas in the presence of Dy^3+^, the DNA–nanopore interactions were barely affected (Fig. S8†), ruling out the possibility of nanopore interior being modified by the coordination of Dy^3+^.

**Fig. 4:**
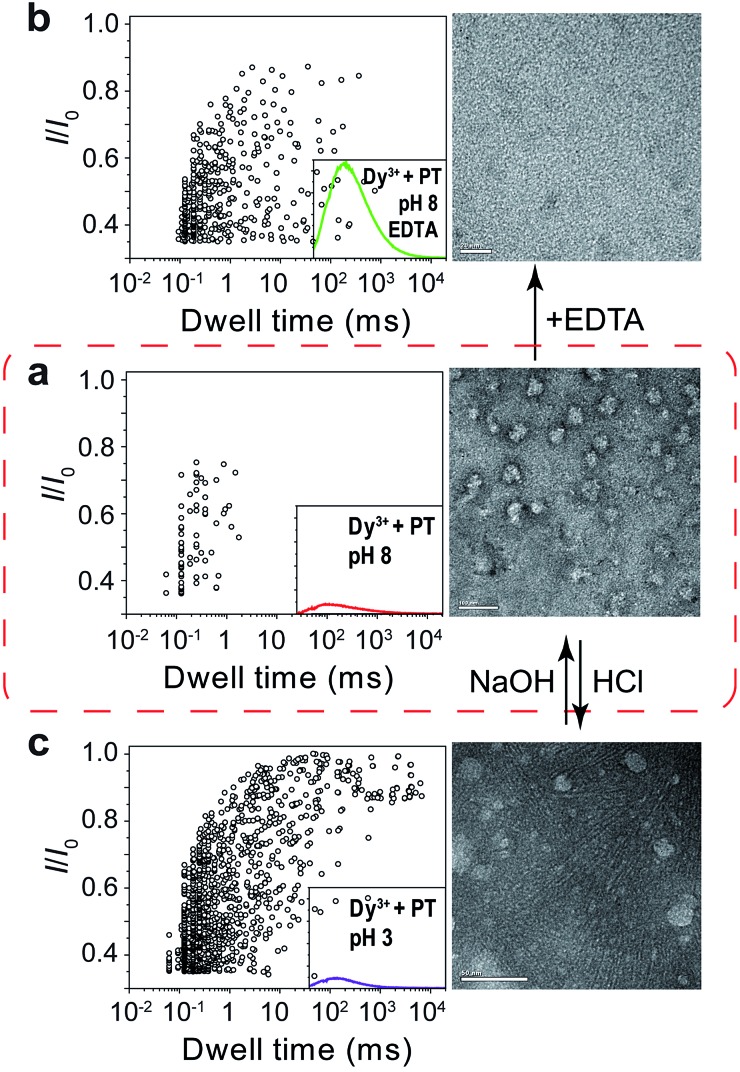
Tuning of the aggregational states of PT molecules. (a) Left: scatter plot showing current blockades (*I*/*I*_0_) *versus* durations of the translocation events of PT (final concentration: ∼75.8 nM) in 1 M KCl, 10 mM Tris and 0.5 μM EDTA, at pH 8.0 in the presence of 50 μM Dy^3+^. The inset shows the emission spectrum of the PT solution for translocation studies (for full spectra and conditions, see Fig. S9†). Right: TEM image of the PT–Dy^3+^ complexes. PT was dissolved in 5 mM potassium phosphate buffer (PB) and 0.5 μM EDTA at pH 8.0, in the presence of 50 μM Dy^3+^. The scale bar is 100 nm. (b) Left: scatter plot of *I*/*I*_0_*versus* dwell time after the addition of 100 μM EDTA in the solution. Right: TEM image of the PT molecules. PT was dissolved in 5 mM PB at pH 8.0, in the presence of 50 μM Dy^3+^ and 100 μM EDTA. The scale bar is 20 nm. (c) Left: scatter plot of *I*/*I*_0_*versus* dwell time after the pH of the solution was adjusted from 8.0 to 3.0. Right: TEM image of the PT aggregates. PT was dissolved in 5 mM PB at pH 3.0, in the presence of 50 μM Dy^3+^. The scale bar is 50 nm. The high resolution TEM images are shown in Fig. S10–12.† Scatter plots were constructed using the data points with *I*/*I*_0_ > 0.35 in a 30 min recording trace. The number of individual single-channel recording experiments *n* = 3.

Next, we sought further evidence to corroborate the above conclusion. The first experiment we conducted was to test the interconversion of different aggregational states of PT molecules. To the solution of Dy^3+^-treated PT at pH 8.0 (Fig. 4a), we added 100 μM EDTA and then recorded the PT translocation events and fluorescence spectrum. It was shown that the frequency of translocation events was recovered to free PT level and the fluorescence of PT was also restored (Fig. 4b). With the same Dy^3+^-treated PT solution, we adjusted the pH of the solution by adding HCl or NaOH. When the pH was lowered to 3.0, we found the frequency of translocation events abruptly increased nearly 60 times which was close to the value of free PT at pH 3.0. However, fluorescence measurements showed that there was barely any difference between before and after the pH alteration (Fig. 4a and b). Then, we performed the second experiment to compare the different aggregational states of PT by taking TEM images of each solution in Fig. 4. It is intriguing that Dy^3+^ indeed crosslinks PT chains which eventually wrap into mesoscale globules (∼50 nm, Fig. 4a), whereas hydrogen-bonding does assembly PT chains into long linear wires (∼50–100 nm, Fig. 4c). These evidences strongly support the results of single-molecule experiments. An additional proof for the formation of globules in Dy^3+^-treated PT was obtained after the solution was kept at 4 °C for a week when orange precipitate was found in the bottom of the tube (Fig. S13†).

**Fig. 5:**
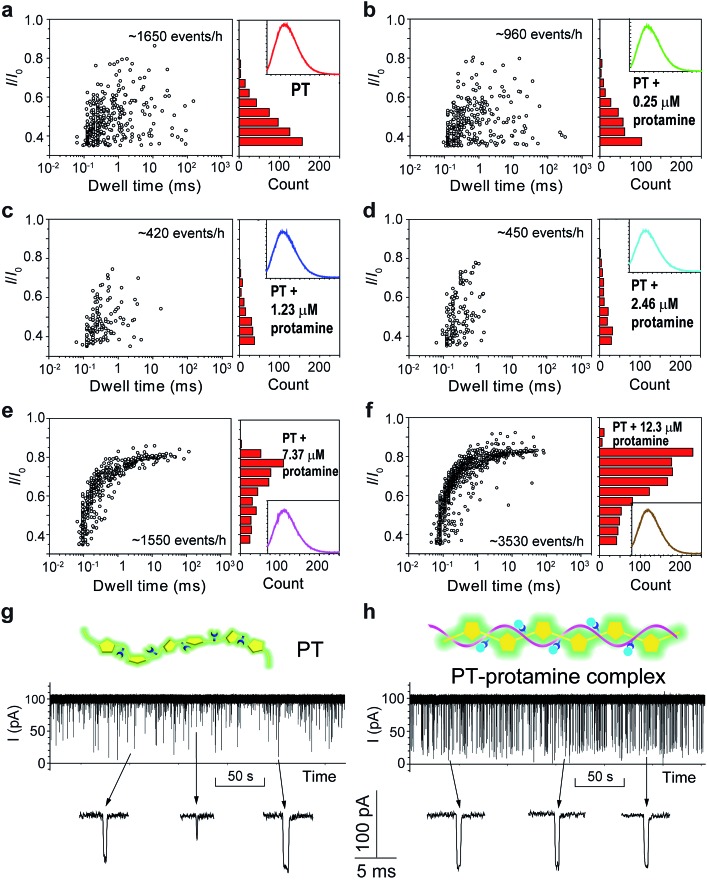
Translocation of PT through αHL in the presence of different concentrations of protamine. (a–f) Left: scatter plots showing current blockades (*I*/*I*_0_) *versus* durations of the translocation events in the presence of 0, 0.25, 1.23, 2.46, 7.37, 12.3 μM protamine, respectively. The frequency of PT translocation is 1650 ± 170, 960 ± 30, 420 ± 25, 450 ± 30, 1550 ± 210, 3530 ± 220 events per h, respectively. Right: histograms of the current blockades of the translocation events in the presence of 0, 0.25, 1.23, 2.46, 7.37, 12.3 μM protamine, respectively. The inset shows the emission spectrum of each PT solution for the translocation studies (for full spectra and conditions, see Fig. S14†). The final concentration of PT is ∼75.8 nM. Both scatter plots and current blockade histograms were constructed using the data points with *I*/*I*_0_ > 0.35 in a 20 min recording trace. (g and h) Translocation traces of PT (g) and PT–protamine complex (h) through αHL. The concentration of PT is ∼75.8 nM and the concentration of protamine is 12.3 μM. The pictures above the traces represent the main conformational polymer species that transit through αHL. Data were acquired in the buffer of 1 M KCl, 10 mM Tris and 0.5 μM EDTA, pH 10, with the transmembrane potential held at +100 mV. The number of individual experiments *n* = 3.

The above results confirmed that different aggregational states of PT molecules could be unambiguously distinguished by PT translocation behavior in αHL when its fluorescence was quenched by the pH tuning or the presence of Dy^3+^. We wondered whether a nanopore sensor could also distinguish different conformational or morphological states of PT while its fluorescence is “ON”. Protamine is one type of arginine-rich proteins that can bind with PT through electrostatic interactions. The changes of PT fluorescence upon protamine binding could be used for sensing protamine.^38^ However, when the sensing experiments were conducted in the buffer containing 1 M KCl, we found that the fluorescence of PT barely changed even when the concentration of protamine reached 12.3 μM (Fig. S14†). This is probably because the electrostatic charge interactions between PT and protamine have been weakened due to the high ionic strength of the electrolyte. Interestingly, when we subjected the PT–protamine complexes to translocation test in αHL, we observed different translocation behaviors along with the varied concentrations of protamine. In the low concentration regime (0–2.46 μM), we observed a marked decrease of the frequency of PT translocation events from ∼1650 events per h to ∼450 events per h when the concentration of protamine was increased from 0 to 2.5 μM (Fig. 5a–d). We attributed these phenomena to the formation of PT–protamine complexes which are too bulky to translocate through αHL, because the PT translocation profile remained similar. However, as the concentration of protamine was further increased to 7.4–12.3 μM, the translocation profile underwent drastic changes (Fig. 5e and f). First, the frequency of translocation events was recovered and doubled when the concentration of protamine was increased to 7.4 and 12.3 μM, respectively. In theory, positively charged protamine binding with PT would decrease the capture rate of the PT–protamine complex (Fig. S15†). This enhanced event frequency means part of the PT (shorter-chain) translocation might have been missed due to the fast translocation speed (*τ* < 100 μs) or some have been removed during data analysis due to their relative shallow current blockage (*I*/*I*_0_ < 0.35). Second, the current blockage (*I*/*I*_0_) was concentrated between 0.6 and 0.8, and the distribution of dwell time (*τ*) also became much narrower (Fig. 5g and h). It is conceivable that as a free polymer in solution, PT can transit through αHL in different conformations such as linear, folded, and globular, resulting in various current blockages and broad distribution of dwell time. While after PT binds with protamine through electrostatic interactions to form a duplex,^47^ the conformation of the PT–protamine complex becomes relatively rigid which is critical for producing uniform current blockages (Fig. 5h). This example illustrates that when PT is in a dispersed state with fluorescence “ON”, its conformational changes can also be discriminated by a nanopore sensor.

## Conclusion

In summary, we have successfully employed nanopore sensors in analyzing different aggregational states of a polythiophene derivative. When the fluorescence of the polythiophene is quenched by pH alteration or addition of certain concentration of Dy^3+^, the UV-vis and fluorescence spectra of the two solutions appear indistinguishable. However, by threading PT molecules through αHL, we could readily discriminate between these two fluorescence “OFF” states. In pH tuning experiments, PT aggregates to form linear wires driven by hydrogen-bonding which produce deep current blockages during PT translocation; while the presence of Dy^3+^ causes PT molecules to crosslink and wrap into mesoscale globules which are too large to enter the nanocavity of αHL. We further substantiated the conclusion by conducting aggregational interconversion experiments and TEM measurements. In addition, we also found that the binding of protamine with PT in 1 M KCl hardly affected the fluorescence “ON” state of PT, but translocation of PT–protamine complexes through αHL afforded distinctly different translocation profiles. These results clearly indicate that nanopore sensors are effective and powerful tools for the analysis of conformational and aggregational changes of conjugated polymers in solution. This may also open new possibilities for the investigation of conformational and aggregational states of biomacromolecules in the context of disease early diagnosis and prognosis. For instance, recent progress on Alzheimer's disease (AD) treatment have provided new findings that are supportive of the amyloid hypothesis in which the deposition of amyloid β-peptide in plaques in brain tissue is the key cause of AD.^48^

## Supplementary Material

Supplementary information
