# Supplementary material for: Revealing different aggregational states of a conjugated polymer in solution by a nanopore sensor
Source: Chem Sci. 2016 May 5;7(8):5287–93. doi: 10.1039/c6sc00296j (PMC6020615; doi:10.1039/c6sc00296j)
Supplement: Supplementary file 1 [file SC-007-C6SC00296J-s001.pdf]

Electronic Supplementary Material (ESI) for Chemical Science.
This journal is © The Royal Society of Chemistry 2016

Supplementary Materials for:

Revealing Different Aggregational States of a Conjugated Polymer in Solution by a Nanopore Sensor

Bingyuan Guo,‡[a] Zhiyi Yao,‡[a] Lei Liu,[a] and Hai-Chen Wu*[ab]

[a]Key Laboratory for Biomedical Effects of Nanomaterials & Nanosafety, Institute of High Energy Physics, Chinese Academy of Sciences, Beijing 100049, China

[b]Beijing National Laboratory for Molecular Sciences, Key Laboratory of Analytical Chemistry for Living Biosystems, Institute of Chemistry, Chinese Academy of Sciences, Beijing 100190, China

*To whom correspondence should be addressed. Email: haichenwu@ihep.ac.cn; Tel: +8610-88235745; Fax: +8610-88235745

‡These authors contributed equally to this work.

Contents

S1. Materials and Methods S2

S2. Voltage selection experiments S4

S3. PT fluorescence changes under different pH values S5

S4. Data selection criterion S6

S5. Interactions between PT and Ca^{2+} in electrolyte buffer solution S7

S6. PT fluorescence changes in the presence of different concentrations of Dy^{3+} S9

S7. DNA control experiments S10

S8. PT fluorescence changes in the interconversion of PT conformations S12

S9. High resolution TEM images S13

S10. Precipitation experiment S16

S11. Fluorescence spectra of PT and PT-protamine complex S17

S12. Translocation of protamine through αHL S18

S13. Supporting information references S19

S1. Materials and Methods

Materials and characterization. 1,2-Diphytanoyl-*sn*-glycero-3-phosphochline (DPhPc) was purchased from Avanti Polar Lipids (Alabaster, AL). Dysprosium (III) nitrate pentahydrate was purchased from Alfa Aesar (99.9%). Protamine sulfate salt from salmon was purchased from Sigma-Aldrich. The molar concentration of protamine was calculated using an average molecular weight of 4070 Dalton. All other chemicals were purchased from Sigma-Aldrich, Alfa Aesar and J&K, and used without further purification. All samples and buffers were prepared in deionised water (Millipore, MA). Fluorescence emission spectra were measured on Horiba Fluorolog-3 FL3-21 spectrometer. UV-Vis spectra were performed on a Hitachi U-3900 spectrometer. High resolution transmission images were obtained on FEI Tecnai 20 transmission electron microscope.

Synthesis of PT. The PT polymer used in this work was synthesized according to the procedure described in one of our previous works.[37] Determination of the average molecular weight of PT by GPC: Mn = 42.6 KDa and Mw = 64.8 KDa (Mn: number-average molecular weight; Mw: weight-average molecular weight; 1 Da = 1 g/mol). The concentration of PT shown in the main text is an average concentration based on the number-average molecular weight unless otherwise stated.

Protein preparation. Wild type αHL-D8H6 monomers (wildtype background with D8H6 tail) were produced by expression in BL21 (DE3) pLysS *Escherichia coli* cells. The monomers were then assembled into homoheptamers on rabbit red blood cell membranes followed by purification with 8% SDS-PAGE as described earlier.[50,51] The purified heptamer protein was conserved in buffer (10 mM Tris-HCl, pH 7.9, 50 mM NaCl) and stored at -70°C.

Buffer preparation. 7.455 g of KCl (99.999%, Sigma-Aldrich) and 0.121 g of Tris (99.9%, Sigma-Aldrich) were dissolved in 80 mL of deionized water (Millipore, MA), then 5 μL, 10 mM EDTA (99.0%, Sigma-Aldrich) stock solution was added. 2 M HCl or 2 M KOH was used to adjust the pH to 3.0, 5.0, 8.0 or 10.0. The solution was diluted with deionized water to 100

mL. The final buffer solutions consist of 1 M KCl, 10 mM Tris and 0.5 μM EDTA at pH 3.0, 5.0, 8.0 or 10.0. PBS used in TEM measurements is phosphate buffered solution.

Single-channel current recording. DPhPc was used to form a synthetic lipid bilayer across an aperture 100-150 μm in diameter in a 25-μm-thick polytetrafluoroethylene film (Goodfellow, Malvern, PA) that divided a planar bilayer chamber into two compartments, *cis* and *trans*. Both compartments contained 1 mL of buffer solution. PT samples were added to the *cis* compartment, which was connected to ground. The *trans* compartment was connected to the head-stage of the amplifier. All experiments were carried out in the buffer of 1 M KCl, 10 mM Tris, 0.5 μM EDTA, at 22.5 ± 2 °C, under the transmembrane potential of +100 mV unless otherwise stated. The pH of the sample solution was adjusted by adding 2 M HCl or NaOH to 3.0, 5.0, 8.0, and 10.0, respectively. Ionic currents were measured by using Ag/AgCl electrodes with a patch-clamp amplifier (Axopatch 200B; Axon instruments, Foster City, CA), filtered with a low-pass Bessel filter with a corner frequency of 10 kHz or 5 kHz and then digitized with a Digidata 1440A A/D converter (Axon Instruments) at a sampling frequency of 100 kHz.

Data analysis. Current traces were analyzed with Clampfit 10.2 software (Axon Instruments). Events were detected using the Event Detection feature, and used to construct scattering plot and amplitude histograms. Origin (Microcal, Northampton, MA) and Clampfit were used for histogram construction, curve fitting and graph presentation. Adobe Illustrator was used for making figures.

S2. Voltage selection experiments

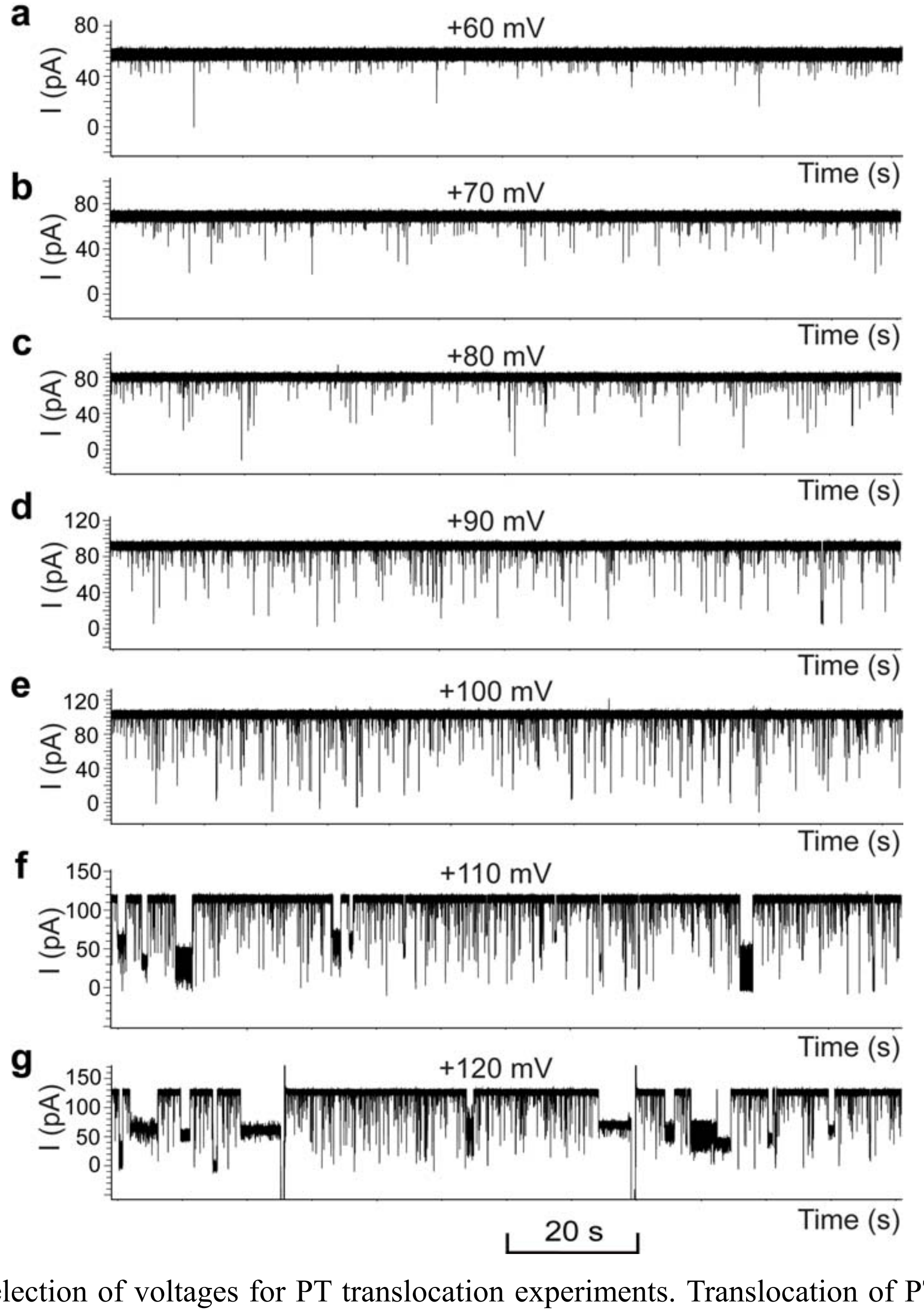

Fig. S1 Selection of voltages for PT translocation experiments. Translocation of PT through αHL was recorded in the buffer of 1 M KCl, 10 mM Tris and 0.5 μM EDTA, at pH 8.0 under the following transmembrane potentials: (**a**) +60 mV; (**b**) +70 mV; (**c**) +80 mV; (**d**) +90 mV; (**e**) +100 mV; (**f**) +110 mV; (**g**) +120 mV. The final concentration of PT is ~75.8 nM. Some unusual long clogging events were observed when the voltage was higher than +110 mV. Therefore, +100 mV was chosen as the trnasmembrane potential for all the following studies.

S3. PT fluorescence changes under different pH values

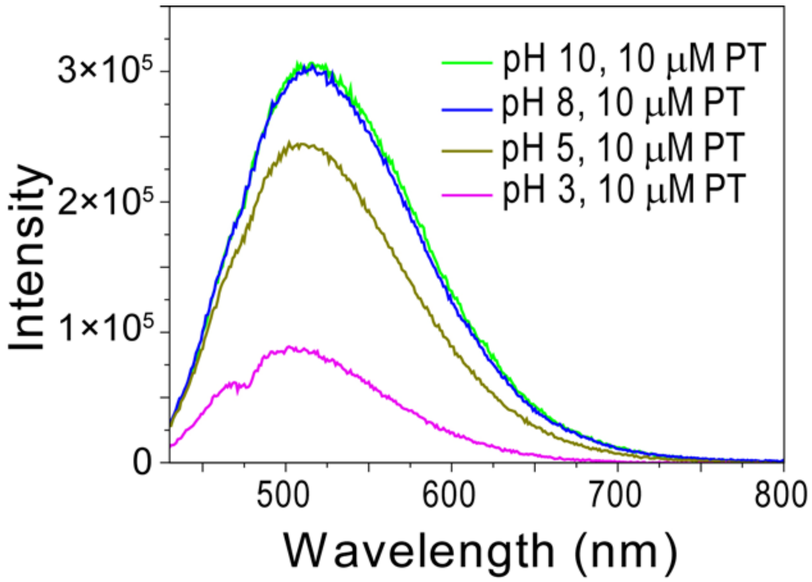

Fig. S2 Emission spectra of PT (~75.8 nM) in the buffer of 1 M KCl, 10 mM Tris and 0.5 μM EDTA under different pH values as indicated. Excitation wavelength λ_{ex} = 410 nm.

S4. Data selection criterion

From comparing with DNA translocation through α-hemolysin nanopore, we could see that the shallow blockades (small I/I_0) with very short durations (< 100 μs), which are independent of the applied potential, correspond to PT molecules colliding with, but not fully translocated through the nanopore.[52] In order to take the meaningful data for statistical analysis, we tried an arbitrary standard (I/I_0) to remove as many as those colliding events. After several attempts, we found that when we used the data points with $I/I_0 > 0.35$ for analysis, we removed most of the colliding events while keeping the majority of the translocation events.

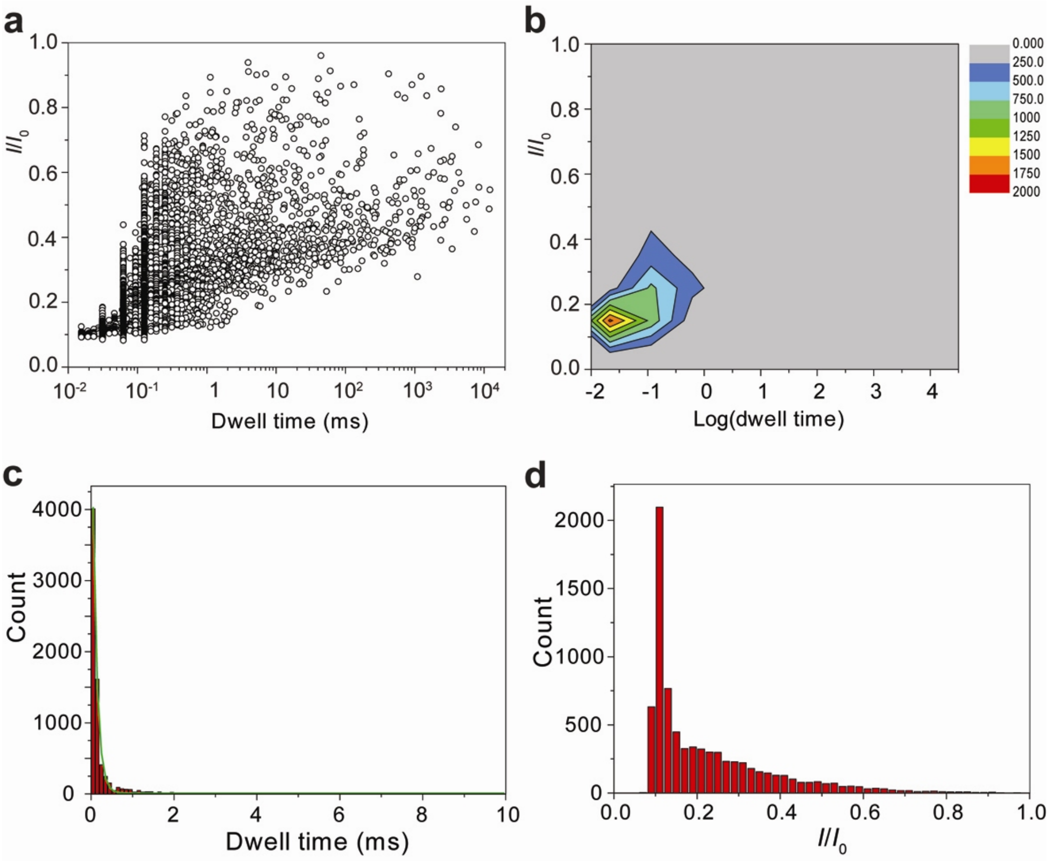

Fig. S3 PT translocation through αHL with all data points. (**a**) Scatter plots showing current blockades (I/I_0) versus durations of the all PT translocation events. (**b**) Density plot showing current blockades (I/I_0) versus log(dwell time) of the all PT translocation events. (**c**) Histogram of the dwell time of the translocation events with the duration < 10 ms (τ = 102 μs). (**d**) Histogram of the I/I_0 distribution of the translocation events. The final concentration of PT is ~75.8 nM. Data were acquired in the buffer of 1 M KCl, 10 mM Tris and 0.5 μM EDTA, pH 8.0.

S5. Interactions between PT and Ca^{2+} in electrolyte buffer solution

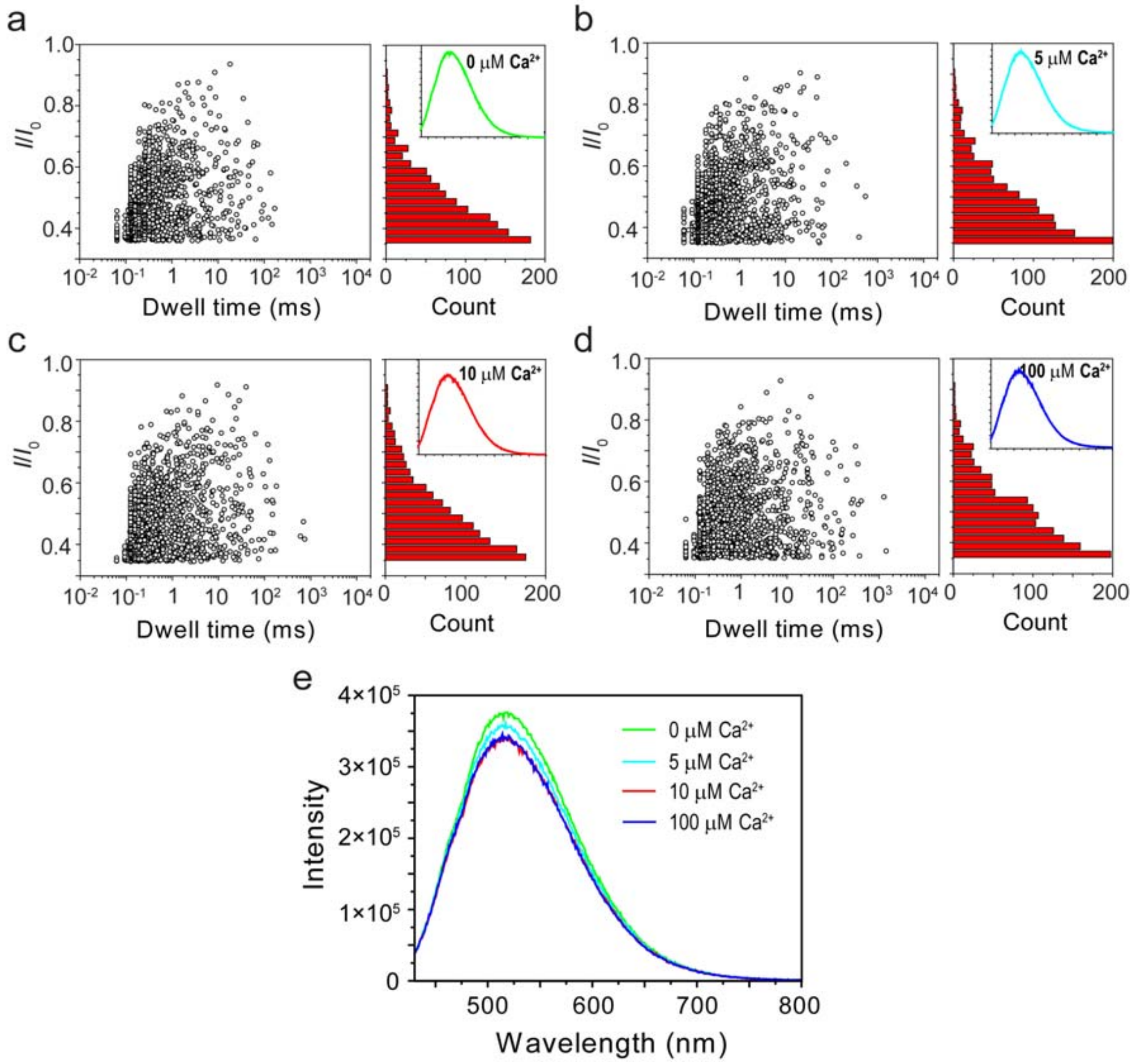

Fig. S4 Translocation of PT through αHL in the presence of different concentrations of Ca^{2+}. (**a**-**d**) Left: scatter plots showing current blockades (I/I_0) versus durations of the translocation events in the presence of 0, 5, 10, 100 μM Ca^{2+}, respectively. The frequency of PT translocation is ~1600 ± 140 events/h for all the recordings. Right: histograms of the current blockades of the translocation events in the presence of 0, 5, 10, 100 μM Ca^{2+}, respectively. The inset shows the emission spectrum of each PT solution for the translocation studies. The final concentration of PT is ~75.8 nM. Both scatter plots and current blockade histograms were constructed using the data points with $I/I_0 > 0.35$. Data were acquired in the buffer of 1 M KCl, 10 mM Tris and 0.5 μM EDTA, pH 10 in the presence of Ca^{2+} (concentrations indicated), with the transmembrane potential held at +100 mV. (**e**) Emission spectra of PT (~75.8 nM) in the buffer of 1 M KCl, 10 mM Tris, pH 10 and 0.5 μM EDTA in the presence of different concentrations of Ca^{2+} as indicated. Excitation wavelength λ_{ex} = 410 nm.

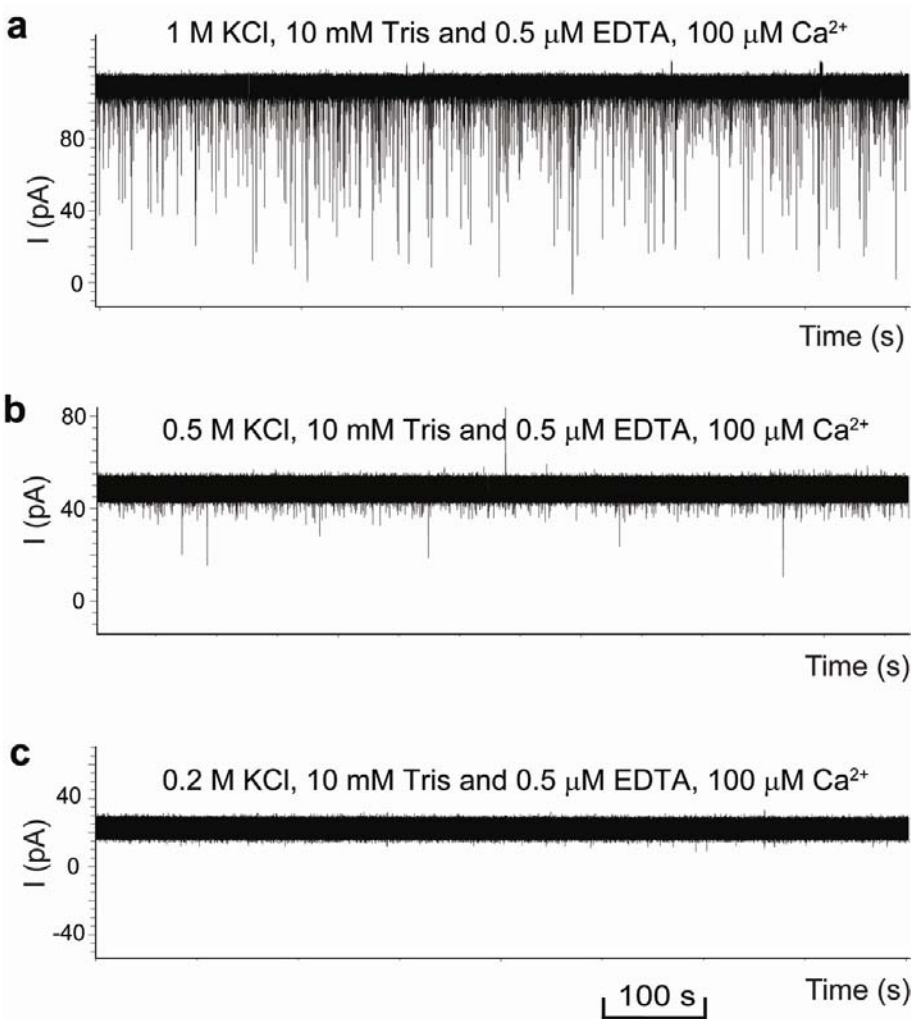

Fig. S5 PT translocation events in the presence of 100 μM Ca^{2+} under different KCl concentrations. (**a**) 1.0 M; (**b**) 0.5 M; (**c**) 0.2 M. The final concentration of PT is ~75.8 nM. Data were acquired in the buffer of 10 mM Tris and 0.5 μM EDTA at pH 10, with the transmembrane potential held at +100 mV.

S6. PT fluorescence changes in the presence of different concentrations of Dy^{3+}

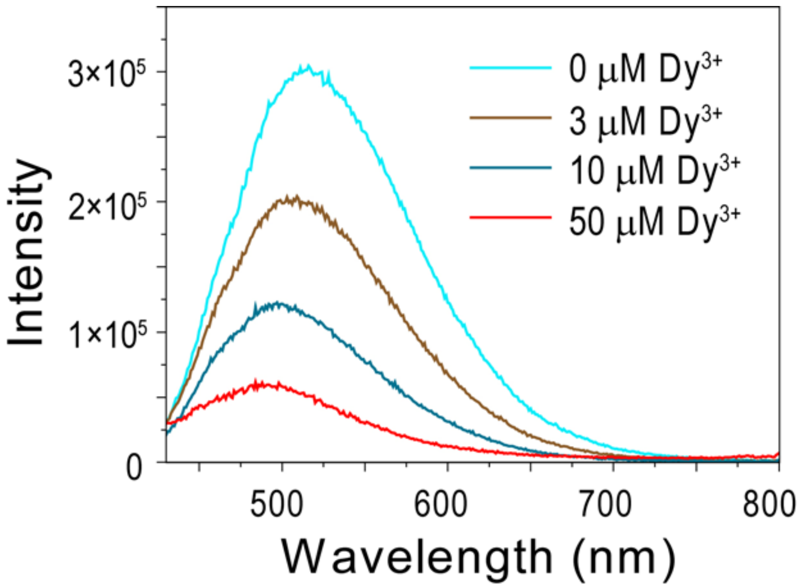

Fig. S6 Emission spectra of PT (~75.8 nM) in the buffer of 1 M KCl, 10 mM Tris and 0.5 μM EDTA, at pH 8.0 in the presence of different concentrations of Dy^{3+} as indicated. Excitation wavelength λ_{ex} = 410 nm.

S7. DNA control experiments

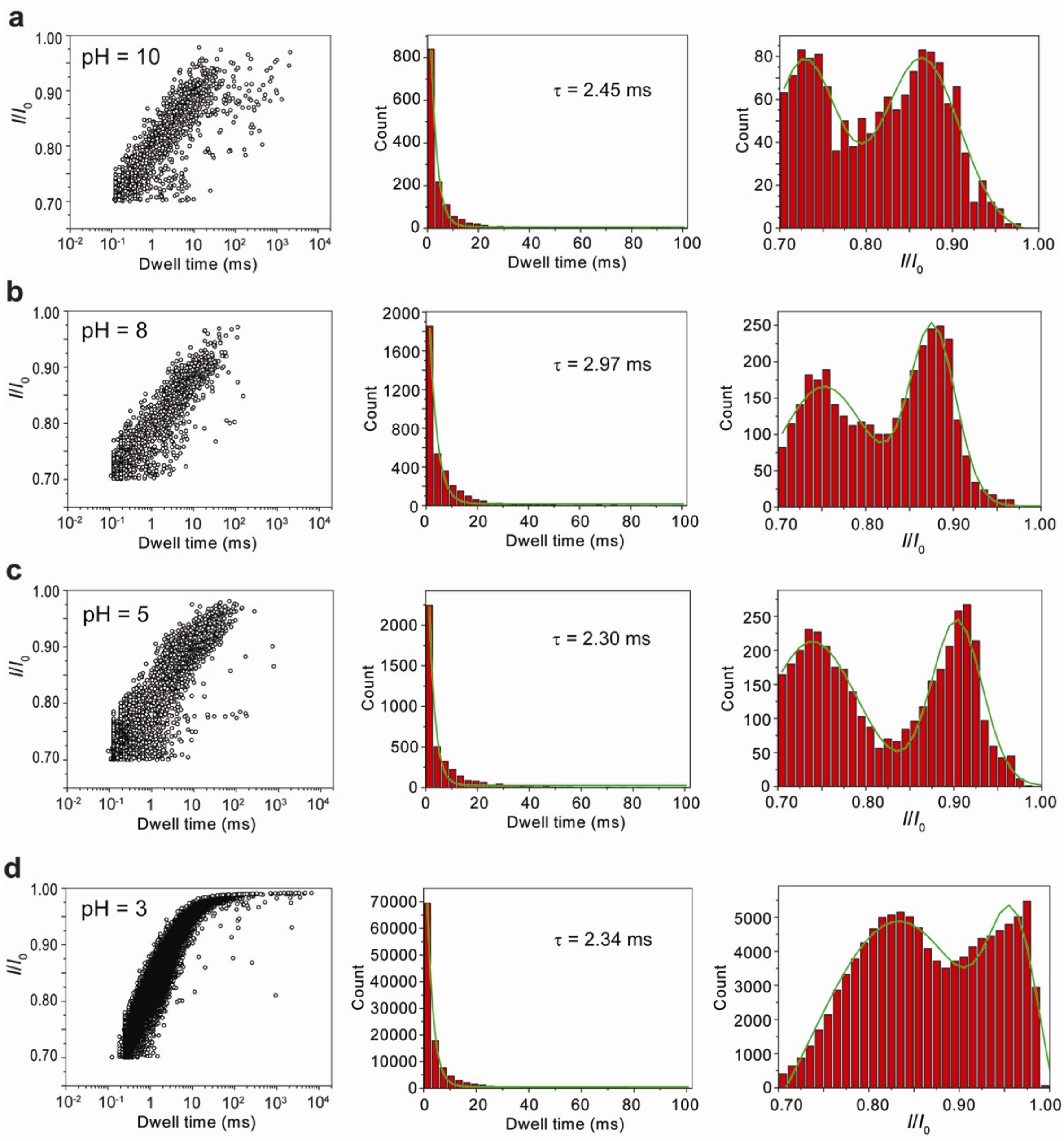

Fig. S7 DNA translocation through αHL in different pH solutions. (**a-d**) Left: scatter plots showing current blockades (I/I_0) versus durations of the translocation events under pH 10.0, 8.0, 5.0, 3.0, respectively. Middle: histograms of the dwell time distribution of the DNA translocation events. Right: histogram of the I/I_0 distribution of the DNA translocation events. The final concentration of poly(dT)$_{30}$ is 500 nM. All the plots were constructed using the data points with $I/I_0 > 0.7$ (ref. 53). Data were acquired in the buffer of 1 M KCl, 10 mM Tris and 0.5 μM EDTA under the pH indicated, with the transmembrane potential held at +100 mV. The number of individual experiments n = 3.

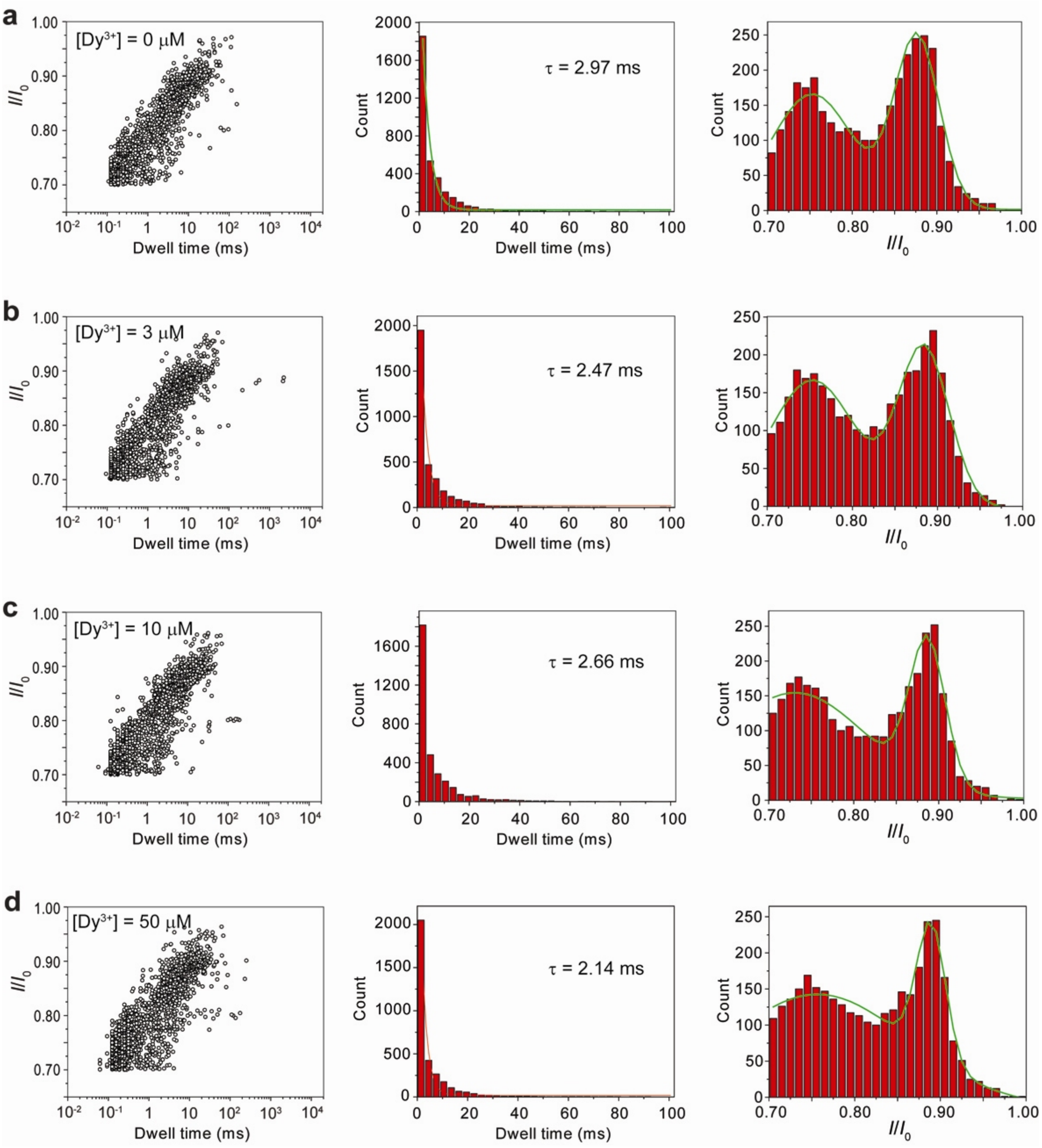

Fig. S8 DNA translocation through αHL in the presence of different concentrations of Dy^{3+}. (**a-d**) Left: scatter plots showing current blockades (I/I_0) versus durations of the translocation events in the presence of 0, 3, 10, 50 μM Dy^{3+}, respectively. Middle: histograms of the dwell time distribution of the DNA translocation events. Right: histogram of the I/I_0 distribution of the DNA translocation events. The final concentration of poly(dT)$_{30}$ is 500 nM. All the plots were constructed using the data points with $I/I_0 > 0.7$. Data were acquired in the buffer of 1 M KCl, 10 mM Tris and 0.5 μM EDTA, pH 8.0 in the presence of Dy^{3+} (concentrations indicated), with the transmembrane potential held at +100 mV. The number of individual experiments n = 3.

S8. PT fluorescence changes in the interconversion of PT aggregations

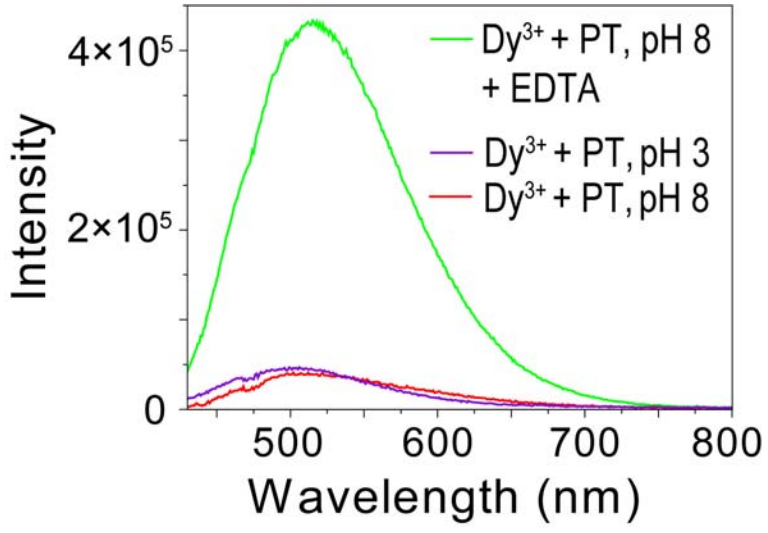

Fig. S9 Emission spectra of PT (~75.8 nM) in the buffer of 1 M KCl, 10 mM Tris and 0.5 μM EDTA, under different conditions as indicated. The final concentration of Dy^{3+} is 50 μM. Excitation wavelength λ_{ex} = 410 nm.

S9. High resolution TEM images

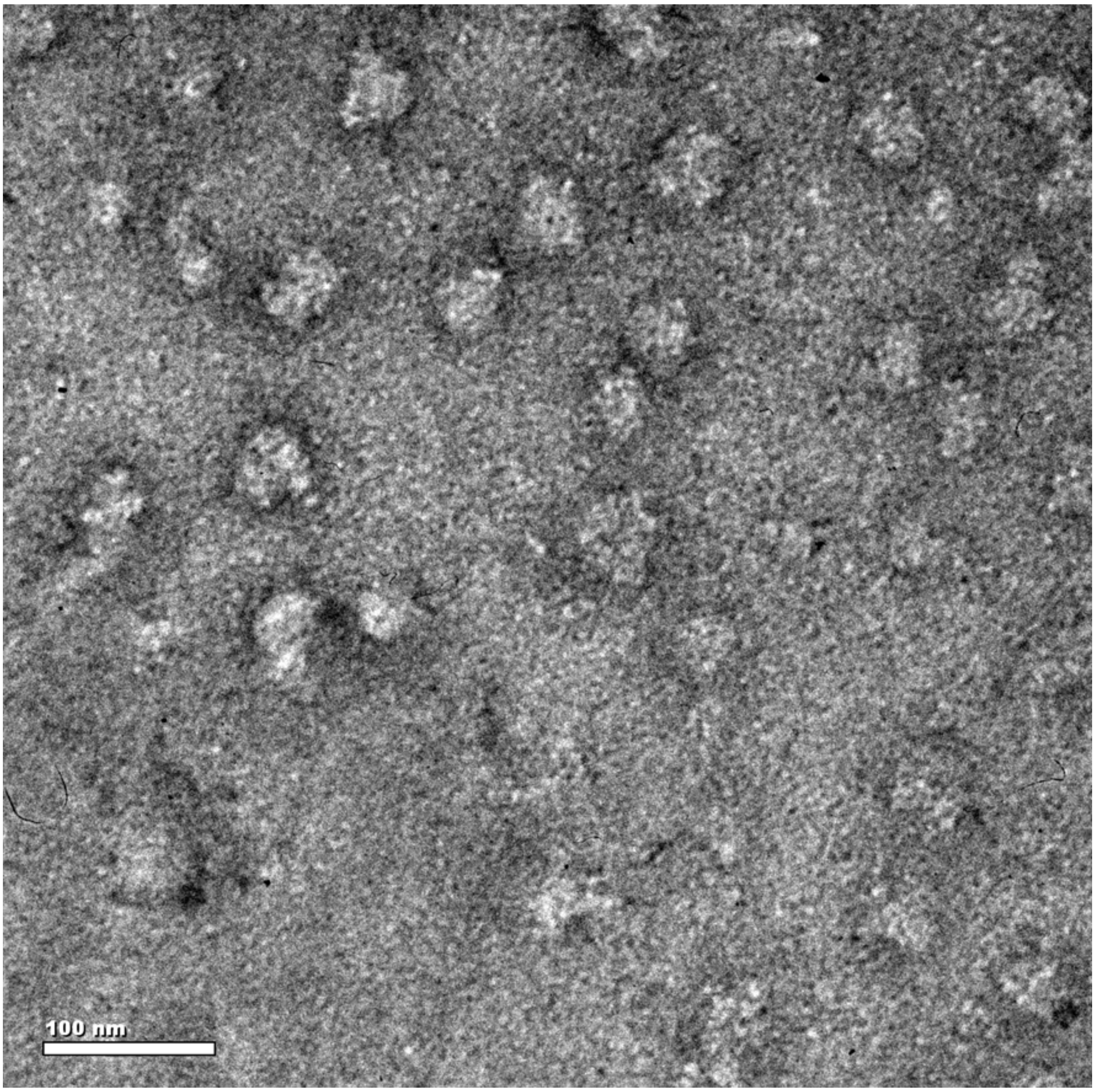

Fig. S10 TEM image of PT-Dy^{3+} complexes. PT was dissolved in 5 mM potassium phosphate buffer (PB) and 0.5 μM EDTA at pH 8.0, in the presence of 50 μM Dy^{3+}. The scale bar is 100 nm.

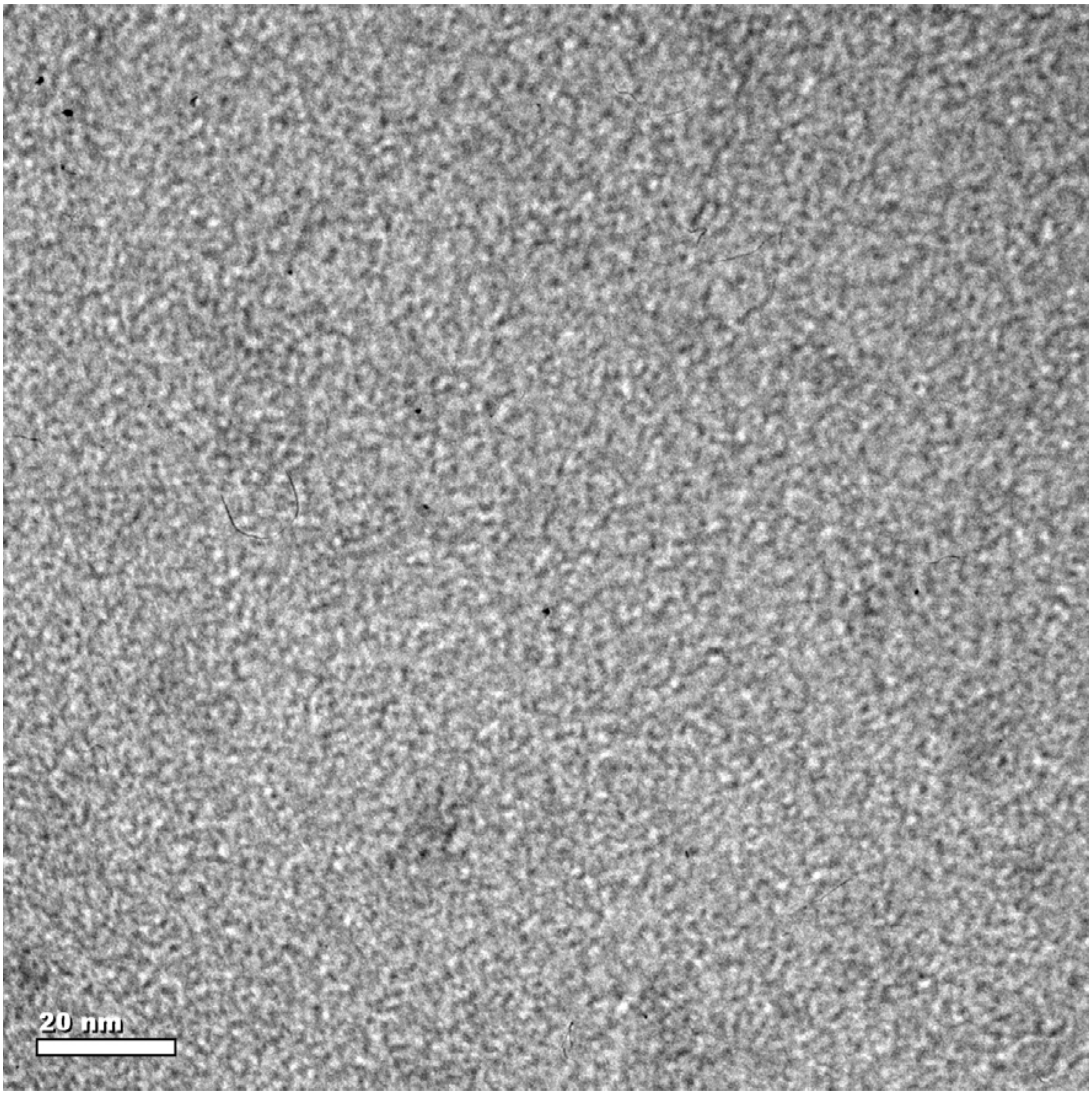

Fig. S11 TEM image of PT molecules. PT was dissolved in 5 mM PB at pH 8.0, in the presence of 50 μM Dy^{3+} and 100 μM EDTA. The scale bar is 20 nm.

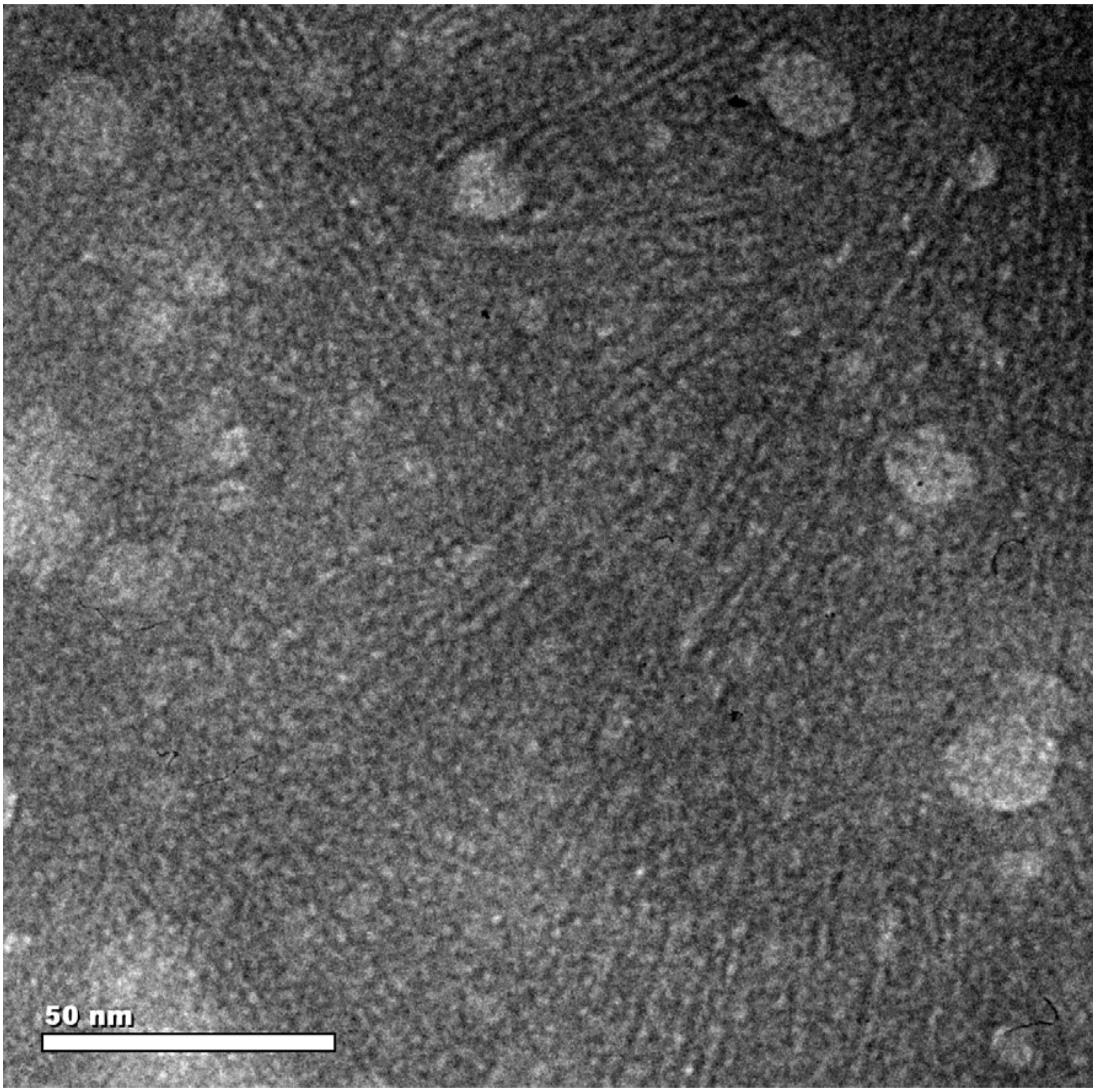

Fig. S12 TEM image of PT aggregates. PT was dissolved in 5 mM PB at pH 3.0, in the presence of 50 μM Dy^{3+}. The scale bar is 50 nm.

S10. Precipitation experiment

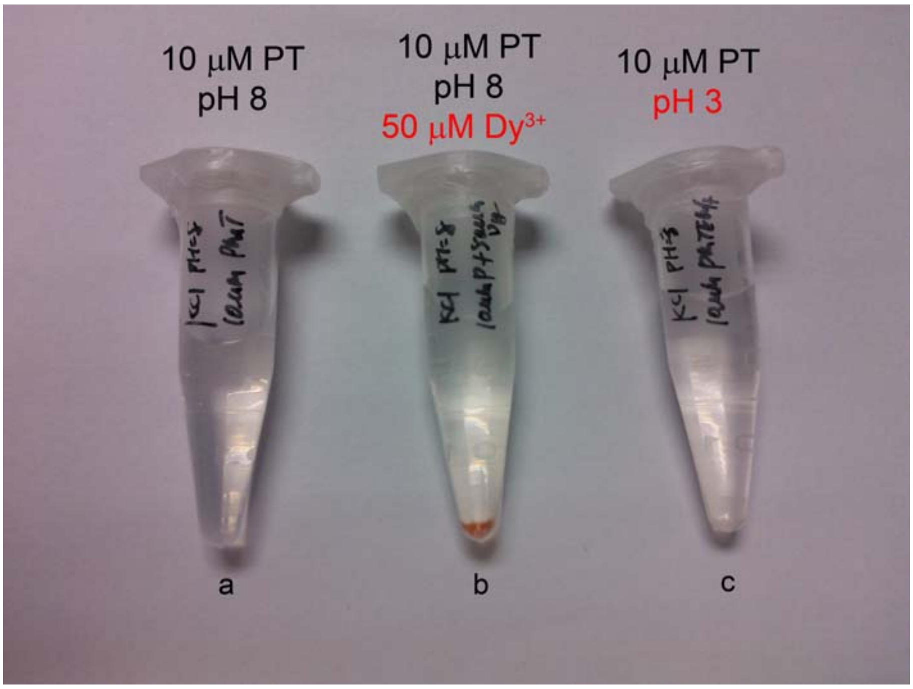

Fig. S13 Precipitation of Dy^{3+}-treated PT solution. The solutions of 75.8 nM PT at pH 8.0 (**a**), 75.8 nM PT mixed with 50 μM Dy^{3+} at pH 8.0 (**b**) and 75.8 nM PT at pH 3.0 (**c**) were kept at 4°C for 7 days. All the solutions were dissolved in the buffer of 1 M KCl, 10 mM Tris and 0.5 μM EDTA. Solutions **a** and **c** were stable during this period but there was orange precipitate in solution **b**.

S11. Fluorescence spectra of PT and PT-protamine complex

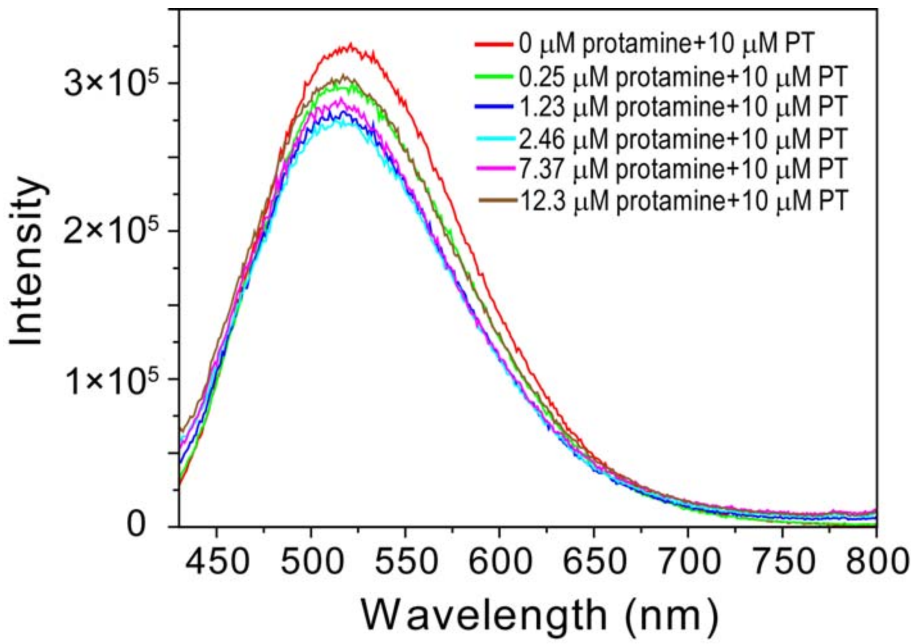

Fig. S14 Emission spectra of PT (~75.8 nM) in the buffer of 1 M KCl, 10 mM Tris and 0.5 μM EDTA, at pH 10.0 in the presence of different concentrations of protamine as indicated. Excitation wavelength λ_{ex} = 410 nm.

S12. Translocation of protamine through αHL

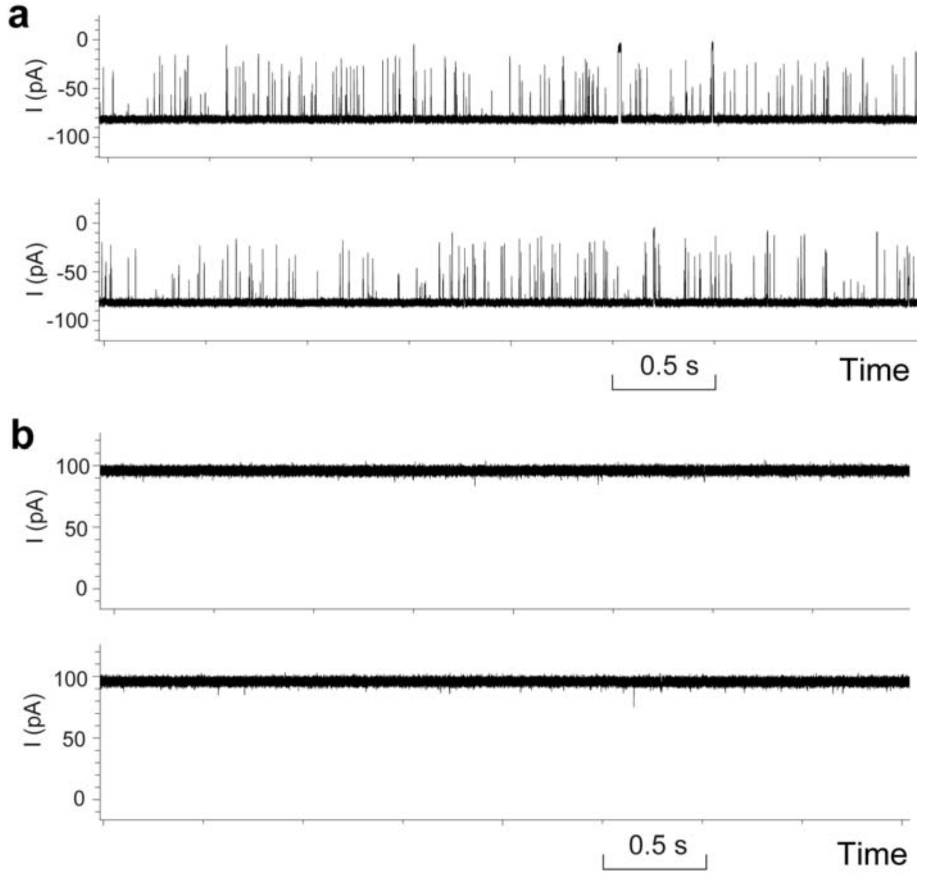

Fig. S15 Translocation trace of protamine through αHL with the transmembrane potential held at (a) -100 mV and (b) +100 mV, respectively. The final concentration of protamine is 12.3 μM. Data were acquired in the buffer of 1 M KCl, 10 mM Tris and 0.5 μM EDTA, pH 10.0. It should be noted that protamine only produces translocation events under applied negative potentials due to its positively charged residues. Therefore, it does not interfere with the single-channel recordings at positive potentials.

S13. Supplementary references

50 S. Cheley, G. Braha, X. F. Lu, S. Conlan and H. Bayley, *Protein Sci.*, 1999, **8**, 1257-1267.

51 S. Wen, T. Zeng, L. Liu, K. Zhao, Y. Zhao, X. Liu, H.-C.Wu, *J. Am. Chem. Soc.* 2011, **133**, 18312-18317.

52 J. J. Kasianowicz, E. Brandin, D. Branton and D. W. Deamer, *Proc. Natl. Acad. Sci. U. S. A.*, 1996, **93**, 13770–13773.

53 A. Meller, L. Nivon, E. Brandin, J. Golovchenko and D. Branton, *Proc. Natl. Acad. Sci. U. S. A.*, 2000, **97**, 1079-1084.
